# The Histopathology of Severe Graded Compression in Lower Thoracic Spinal Cord Segment of Rat, Evaluated at Late Post-injury Phase

**DOI:** 10.1007/s10571-021-01139-7

**Published:** 2021-08-19

**Authors:** Jana Fedorova, Erika Kellerova, Katarina Bimbova, Jaroslav Pavel

**Affiliations:** Department of Neurodegeneration, Plasticity and Repair, Institute of Neurobiology, Biomedical Research Center of the Slovak Academy of Sciences, Soltesovej 4-6, 040 01 Kosice, Slovakia

**Keywords:** Spinal cord injury, Severe compression, Functional recovery, Motor deficit, Histopathology, Rat

## Abstract

Spontaneous recovery of lost motor functions is relative fast in rodent models after inducing a very mild/moderate spinal cord injury (SCI), and this may complicate a reliable evaluation of the effectiveness of potential therapy. Therefore, a severe graded (30 g, 40 g and 50 g) weight-compression SCI at the Th9 spinal segment, involving an acute mechanical impact followed by 15 min of persistent compression, was studied in adult female Wistar rats. Functional parameters, such as spontaneous recovery of motor hind limb and bladder emptying function, and the presence of hematuria were evaluated within 28 days of the post-traumatic period. The disruption of the blood-spinal cord barrier, measured by extravasated Evans Blue dye, was examined 24 h after the SCI, when maximum permeability occurs. At the end of the survival period, the degradation of gray and white matter associated with the formation of cystic cavities, and quantitative changes of glial structural proteins, such as GFAP, and integral components of axonal architecture, such as neurofilaments and myelin basic protein, were evaluated in the lesioned area of the spinal cord. Based on these functional and histological parameters, and taking the animal’s welfare into account, the 40 g weight can be considered as an upper limit for severe traumatic injury in this compression model.

## Introduction

Despite the many experimentally promising therapeutic approaches invented in recent decades, there is no reliable, effective and clinically accepted treatment currently available for disabled patients after spinal cord injury (SCI). Therefore, intensive research into spinal cord trauma is still necessary, particularly for a better understanding of the destructive secondary pathophysiological mechanisms that are ongoing in the injured spinal cord after the primary mechanical insult. A wide range of SCI models are used to gain a detailed understanding of the complexity of human SCIs and to investigate the efficacy of possible therapeutic interventions under controlled conditions in terms of the animal utilized, spinal cord segmental level and injury mechanisms. An optimal experimental model of SCI should meet several key criteria: (1) the sufficient simulation of a human clinical SCI, (2) availability, (3) adequate reproducibility, and (4) the capability of creating different degrees of severity and functional outcomes (Nardone et al. 2017). However, none of experimental models can encompass all aspects of injuries seen in clinical practice.

In spite of some dissimilarities, rat models of SCI demonstrate similar functional, morphological and electrophysiological results to those observed in human SCIs (Kjell and Olson 2016; Metz et al. 2000), and are therefore used frequently in pre-clinical studies. Although the cervical region is most commonly injured in human SCIs, a systematic review of relevant literature revealed that approximately 81% of experimental SCIs are performed at the thoracic spinal level (Sharif-Alhoseini et al. 2017) probably for reasons of feasibility and animal welfare. Statistically, most human SCIs are caused by compression or contusion of the spinal cord (Anwar et al. 2016); therefore, these type of injuries are considered as relevant and commonly used models in experimental studies (Alizadeh et al. 2019). Whereas contusion causes an acute and transient injury to the spinal cord, a compression injury is characterized by spinal cord compression over an extended time period. In fact, some of the compression models are contusion-compression models that include an acute initial impact to the spinal cord followed by its persistent compression. Experimental compression injuries can be undertaken with aneurism clip (Poon et al. 2007), calibrated forceps (McDonough et al. 2015), or a tiny balloon inserted and inflated in the spinal canal (Vanicky et al. 2001). Clip and forceps compression models usually provide bilateral lesions of the spinal cord, whereas unidirectional forces occur during common weight- and balloon-induced compression. A major disadvantage of many compression-induced SCI models is the absence of recorded biomechanical parameters characterizing an initial insult. According to our experience and that of others (Cheriyan et al. 2014), the dural sac involving the spinal cord surrounded with cerebrospinal fluid is so flexible that even a force-controlled impact inducing a contusion injury or balloon-inflation in the spinal canal is not always properly applied on a given spinal segment as desired; subsequently, an inconsistent parenchymal injury and functional deficit may occur. Therefore, we decided to use a weight-compression model of the spinal cord in our experimental studies. The main advantage of the selected traumatic model is that it can generate a controlled and symmetrically centered lesion of the spinal cord at the desired segmental level. Thus, it can limit a high variability in histological and functional outcomes, subsequently creating a marked increase in the possibility of detecting the ongoing cellular and molecular events leading to spinal cord tissue destruction. Although the requirement of laminectomy can be considered as a major limitation, it may help to decrease a secondary compression induced by post-traumatically elevated intraspinal pressure. Thus, critical requirements for performing a standard compression-induced SCI are a minimal standardized size of dorsal laminectomy, and careful stabilization of vertebral columns before the injury.

In SCI research, rodents are limited by their spontaneous recovery of lost motoric functions due to structural plasticity and local organization of spared axons, e.g., collateral sprouting, which is not considered to occur in humans (Friedli et al. 2015; Kjell and Olson 2016). A spontaneous recovery is relatively fast after very mild/moderate SCIs in rats, and this may complicate a reliable evaluation of potential therapeutic approaches (Nardone et al. 2017). On the contrary, improvement of functional outcomes is markedly limited; thus, the effectiveness of prospective treatments can be masked in very severe injuries. Moreover in the human clinic, the most devastating SCIs can be classified as a “severe” rather than “moderate” degree of injury performed in most rodent experimental studies (Blight and Tuszynski 2006). In order to achieve a well-defined lesion in the rat spinal cord based on histological and functional parameters, the main goal of our study was to characterize the severity-dependent pathology of a weight-compression model at the late post-injury period that exhibits a low and relative stable degree of spontaneous regeneration.

## Materials and Methods

### Animals and Experimental Design

Adult female Wistar rats (260–320 g weight, 3–4 months of age) were used for experimental studies. They were randomly divided into four groups: (1) intact control (*n* = 10); (2–4) SCI induced by 30 g, 40 g or 50 g compression at the Th9 segmental level, lasting 15 min (*n* = 23 per group). Experimental animals were housed in standard cages with a lid to hold food and a water bottle for ad libitum eating and drinking at a permanent temperature of 22 °C and relative humidity 55% on a 12 h light/dark cycle.

### Surgery and SCI

All surgical steps were minimally invasive and performed under aseptic conditions. Prior to surgery, the experimental animals were gently weighed and then deeply anesthetized with inhalation gas, 1.0–1.5% isoflurane (Chemical Iberica, PV, Salamanca, Spain) delivered via facemask. To prevent hypothermia, a stable core body temperature (37 °C) was monitored using a flexible rectal thermometer and maintained by a homeothermic heating pad (ATC1000, WPI, Sarasota, FL, USA) throughout surgery. After disinfection of the surgery site with betadine (EGIS Pharmaceuticals PLC, Hungary), a longitudinal skin incision was made, the paraspinous muscles retracted laterally and minimal dorsal laminectomy performed to expose the Th9 spinal segment. Thereafter, the backbone was slightly elevated and fixed by clamping with chirurgical forceps in a stereotaxic frame. The compression-induced SCI was performed as previously described (Fedorova and Pavel 2019). Briefly, a steel rod terminating in a plastic arch-shaped impactor (rectangle base, 2.5 × 2.0 mm, w × l) was symmetrically positioned with the micromanipulator closely above the exposed Th9 segment and then slowly lowered perpendicularly until its weight fully rested on the particular spinal segment. The custom-made impactor was generated from ABS-M30i biocompatible material using a uPrint SE 3D printer (Stratasys, MN, USA). After 15 min of spinal cord compression, the impactor was removed and the wound sutured with sterile silk (USP 4/0, Chirana, CR) in anatomical layers. Immediately after surgery, the experimental animal with lesioned spinal cord was injected with antibiotic, Amoksiklav (12.5 mg/kg, s.c., every 24 h for 3 days) (Sandoz Pharmaceutical, Germany), and analgesic, Novasul (500 mg/kg for 24 h, i. m.) (RichterPharma, Austria), and labeled with permanent marker on the tail for further identification. Sterile core temperature saline solution (5 mL) (Bieffe Medital S.P.A., Italy) was injected subcutaneously to prevent dehydration and to compensate blood loss during surgery. Subsequently, after recovery from anesthesia, rats were housed individually in cages with soft absorbent bedding, moved back to the animal facility, and postoperatively monitored on a daily basis throughout the 28-day post-traumatic period.

### Behavioral Locomotor Testing

The recovery of hind limb motor function was evaluated by the standardized locomotor rating test according the Basso, Beattie, and Bresnahan (BBB) (Basso et al. 1995), which represents a 21 points scale, where a score of 0 means complete paralysis with no observable hind limb movement and a score of 21 represents complete physiological mobility. The BBB score reflects the animal’s hind limb joint movements, stability, paw placement, stepping, forelimb-hind limb coordination, and trunk position. Behavioral testing was performed for 5 min in an open field, once per 2 days, up to 28 days post-injury, by two trained evaluators, independently and without knowing the compression force that the rat had received, in order to eliminate possible testing subjectivity. Right and left hind limb scores were then averaged and final results expressed as mean ± standard error of the mean (SEM).

### Evaluation of Bladder Function

During the post-traumatic period characterized by bladder areflexia, the animals´ bladders were emptied daily by manual expression (Crede’s maneuver) until the voiding reflex completely returned. Female rats were preferred due to their shorter and wider urethra, which allows more effective and easier manual bladder emptying. A two-point score scale was established for the evaluation of urinary bladder function, as follows: 0 point represents a complete loss of bladder function (necessity of manual bladder expression), 1 point means normal physiological bladder function. In addition, we also observed the eventual presence of hematuria or pyuria.

### Tissue Processing

**Fig. 1:**
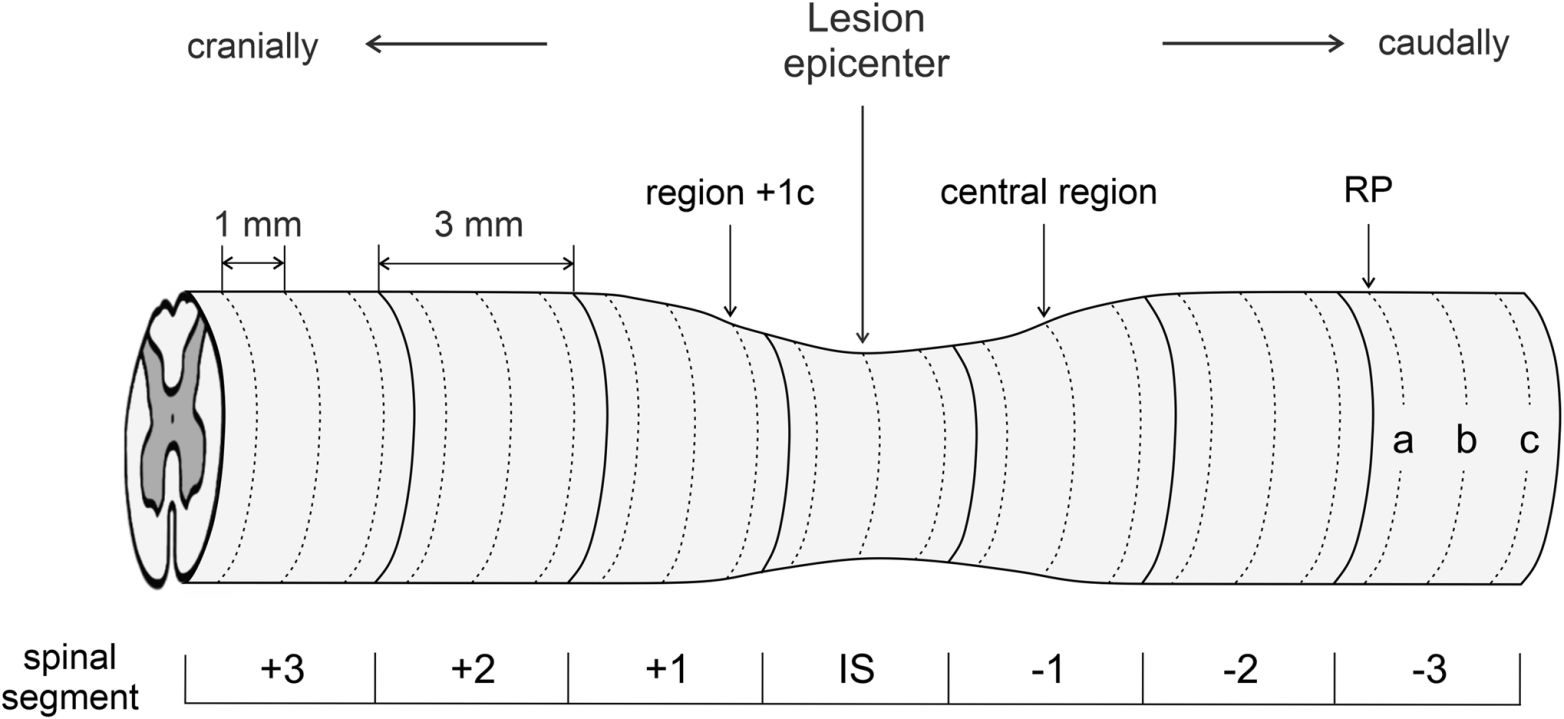
Schematic illustration describing spinal cord sampling. Generally, for all experimental procedures used, the spinal cord was sliced into 3 mm segments, including the injury site (IS) and three cranial (+ 3, + 2, + 1) and three caudal (− 1, − 2, − 3) spinal segments. Non-fixed spinal cord segments were used for biochemical analysis; however, for other experimental procedures, the consecutive sections at regular 1 mm intervals, defined as spinal regions from each segment, were cut and analyzed. Caudal region − 3a was used as a reference point (RP) for calculation of correction factors in histological analyses. For some illustrative purposes, only sections from the central region of each spinal segment were used. *IS* injury site

The spinal cord tissue sampling and processing was dependent on subsequent experimental analysis. Transcardial perfusion after animal sedation with an overdose of Exagon i.p. (RichterPharma, Austria) was performed first with standard saline buffer (pH 7.9) and then by 4% paraformaldehyde prepared in 0.1 M phosphate buffered saline (PBS, pH 7.4). The spinal cord involving the epicenter of the injury and the adjacent caudal and cranial regions were exposed and removed from the spinal canal for further histological staining and immunohistochemical labeling. Only transcardial perfusion with saline was performed for the study of the blood-spinal cord barrier (BSCB) disruption by extravasated Evans blue dye. The spinal cord was then transversely sliced into 3 mm segments using a spinal cord coronal matrice (Electron Microscopy Sciences, PA, USA) (Fig. 1). The caudal ending of each spinal segment was tagged with permanent marker to determine proper cranio-caudal orientation and post-fixed in the same fixative overnight. Following post-fixation, tissue blocks were transferred into graded cryoprotective medium (20% and 30% sucrose in 0.1 M PBS) at 4 °C until saturation. As a consequence of biochemical analyses that require tissue samples without any chemical contamination, animals predetermined for Western blot analyses were decapitated 28 days after the SCI. Following decapitation, the spinal cord was exposed, removed from the spinal canal and sliced into 3 mm blocks as described previously. All collected tissue was then frozen in liquid nitrogen and stored at − 80 °C until further analysis.

### Histological Staining

Cryoprotected spinal cord segments were mounted on specimen holders, covered with M-1 embedding matrix (Thermo Fischer Scientific, MA, USA), frozen in Leica CM1850 cryostat (Leica Biosystems, Germany), cut into 25 µm sections from each region at 1 mm intervals (Fig. 1), and mounted on SuperFrost Plus adhesion microscope slides. The extent of tissue damage was analyzed using Luxol fast blue (to stain and observe myelin/myelinated axons) counterstained with Cresyl violet (to stain neuronal Nissl substance) (LFB/CV) according to a standard protocol. Briefly, the spinal sections were treated with 0.1 M PBS for 10 min, 70% ethanol for 120 min, and incubated with 0.1% LFB solution overnight at room temperature (RT). Next day, sections were washed with distilled water to remove excess stain, treated with 0.1 M PBS for 30 s, and then differentiated in 0.05% lithium carbonate solution for 20 s and 40% ethanol for 20 s until the gray and white matter could be distinguished. Subsequently, sections were again rinsed in distilled water and counterstained with 0.2% CV solution for 10 min. After another washing in distilled water, the sections were dehydrated by passing through increasing concentrations of ethyl alcohol (from 80 to 100%), cleared in xylene, and coverslipped using Entellan mounting medium (Merck KGaA, Germany). Stained spinal cord sections were automatically scanned by an Apperio AT2 digital scanner (Leica Biosystems, Germany) at ×20 magnification.

### Histological Image Analysis

The integrity of spared spinal cord tissue and the extent of damage were analyzed according to our previously published histological quantification method (Fedorova and Pavel 2019), which successfully overcomes negative factors resulting from post-traumatic spinal cord shrinkage, such as an underestimation of tissue loss or overestimation of tissue sparing, as well as markedly eliminating a subjective evaluation. Similarly, five intact spinal cords were cut into 25 µm thick sections in the same manner as mentioned above, mounted on SuperFrost Plus slides and stained with LFB/CV in order to establish the correction factors necessary for prediction of pre-injury cross-sectional white and gray matter area in each region along the spinal cord. Based on the cranio-caudal extent of tissue damage, the most appropriate reference point, defined as the most proximate caudal region unaffected by the traumatic injury, was determined as region − 3a (Fig. 1). The correction factors shown in Table 1 were then calculated as the ratio of the cross-sectional area of white or gray matter at each region along the spinal cord to the white or gray matter area at the reference point for each individual animal. The final correction factors were expressed as mean ± SEM. The predicted pre-injury area occupied by white or gray matter in each region along the lesioned spinal cord was calculated as a product of the correction factor and the cross-sectional area of the reference point (region − 3a).

**Table 1:** Correction factors designed for estimation of white and gray matter cross-sectional area at each region along the lesioned spinal cord

Segment	Region	Mean correction factor ± SEM	
		White matter	Gray matter
+ 3	*a*	0.92 ± 0.05	0.69 ± 0.03
	*b*	0.91 ± 0.05	0.70 ± 0.04
	*c*	0.93 ± 0.04	0.73 ± 0.01
+ 2	*a*	0.91 ± 0.04	0.73 ± 0.03
	*b*	0.90 ± 0.05	0.71 ± 0.02
	*c*	0.94 ± 0.04	0.76 ± 0.04
+ 1	*a*	0.95 ± 0.05	0.69 ± 0.03
	*b*	0.91 ± 0.04	0.69 ± 0.02
	*c*	0.93 ± 0.03	0.73 ± 0.01
IS	*a*	0.94 ± 0.04	0.75 ± 0.02
	*b*	0.93 ± 0.05	0.75 ± 0.03
	*c*	0.96 ± 0.04	0.88 ± 0.02
− 1	*a*	0.98 ± 0.04	0.83 ± 0.01
	*b*	0.99 ± 0.06	0.89 ± 0.04
	*c*	1.02 ± 0.04	0.88 ± 0.04
− 2	*a*	1.01 ± 0.02	0.96 ± 0.02
	*b*	1.01 ± 0.03	0.98 ± 0.02
	*c*	1.03 ± 0.03	0.97 ± 0.03
− 3	*a*	1.00 ± 0.00	1.00 ± 0.00
	*b*	1.03 ± 0.02	1.06 ± 0.02
	*c*	1.07 ± 0.01	1.13 ± 0.03

*Values are expressed as mean ± SEM of 5 intact experimental animals, measured individually

**Fig. 2:**
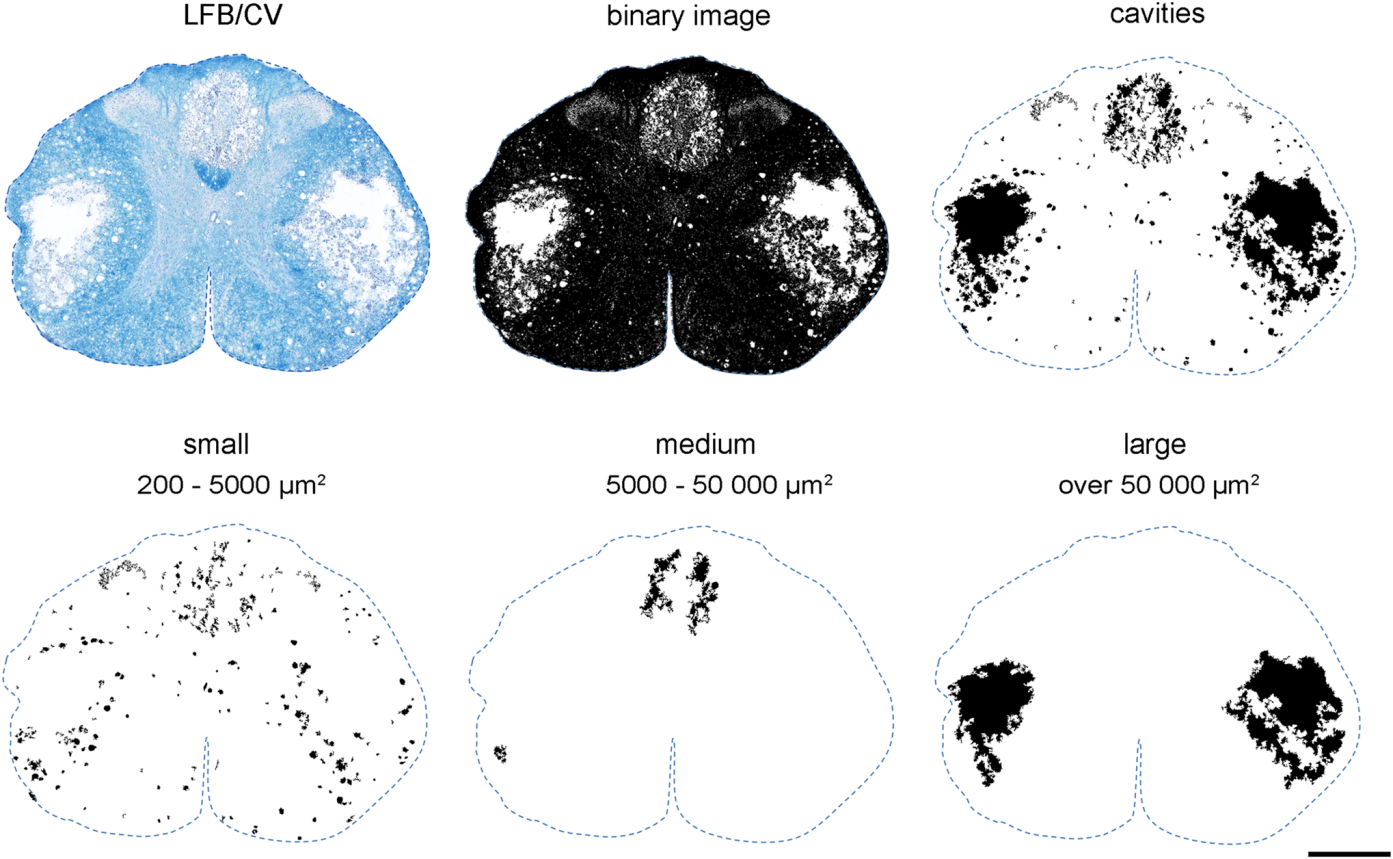
Histological demonstration of spared tissue (shown as binary image) and cystic cavities (shown as inverted binary image) visualized after LFB/CV staining, and classification of cystic cavities into three subclasses depending on actual size (lower row): small (200–5000 µm^2^), medium (5000–50,000 µm^2^) and large (over 50,000 µm^2^). Quantitative measurement was performed automatically using NIH ImageJ software. Scale bar 600 µm

All acquired digital images were quantitatively analyzed using NIH ImageJ Software. Following the scale calibration, RGB digital images were transformed to 8-bit grayscale, the Huang automatic threshold was then applied to satisfactorily separate the LFB/CV stained spinal cord from background, and binary images then created (Fig. 2). Subsequently, the area of white and gray matter was measured from the binary image in each region and the amount of spared tissue calculated by the following formula:

$${\text{spared}}\;{\text{white}}/{\text{gray}}\;{\text{matter}}\;{\text{tissue }}\left( \% \right) = \frac{{{\text{cross-sectional}}\;{\text{white/gray}}\;{\text{matter}}\;{\text{area }}\left( {{\text{mm}}^{{2}} } \right)}}{{{\text{predicted}}\;{\text{cross-sectional}}\;{\text{white/gray}}\;{\text{matter}}\;{\text{area }}\left( {{\text{mm}}^{{2}} } \right)}} \times 100$$

The cystic cavities were visualized from the inverted binary image, automatically numbered, and the occupied area calculated. Numbered cystic cavities were then divided into three groups that were established depending on their actual size: [1] small cavities (from 200 to 5000 µm^2^), [2] medium cavities (5000–50,000 µm^2^), [3] large cavities (over 50,000 µm^2^) (Fig. 2). All obtained histological data were finally presented as mean ± SEM.

### Evaluation of BSCB Disruption

Disruption of BSCB integrity was analyzed by an adapted Evans blue dye quantification method, optimized for limited rat tissue samples (Wang and Lai 2014). Evans blue dye prepared as 4% solution in 0.9% saline (pH 7.4) was injected into the tail vein of deeply anesthetized experimental animal (*n* = 6/group) as a single bolus dose 2 mL/kg. The administration of Evans blue was performed 24 h after SCI, when maximal blood–brain barrier permeability was determined (Figley et al. 2014). Prior to spinal cord isolation, 120 min after Evans blue injection, each rat was transcardially perfused with 0.9% sterile saline (pH 7.4) to get rid of the dye and circulating blood from the vascular network. The isolated spinal cord was then sliced in the standard way into 3 mm segments, as suggested in Fig. 1, and weighed. Separated spinal segments from four animals per group were homogenized in 50% trichloroacetic acid (TCA) at 1:3 ratio (weight:volume), and centrifuged at 10,000×*g* RCF for 20 min. Thereafter, supernatants were diluted with 95% ethanol in a 1:3 ratio, thoroughly mixed, and added to a 96-well microplate for florescence spectroscopy measurement at 620 nm/680 nm using Synergy 2 reader (BioTek, USA). The concentration of extravasated Evans blue was expressed as mean ± SEM. Spectrofluorometric analysis was verified and supplemented by direct fluorescence visualization of extravasated Evans blue dye in 25 µm sections prepared from each spinal region (*n* = 2 animals per group) using an Olympus BX51 fluorescence microscope (Olympus Life Science, Tokyo, Japan) at ×10 magnification.

### Western Blot Analysis

Tissue samples (*n* = 5 animals per group) were homogenized by a Minilys personal homogenizer (Bertin Instruments, France) in RIPA lysis and extraction buffer (Sigma-Aldrich, MO, USA) containing protease inhibitor cocktail (Roche Diagnostics, Switzerland). Homogenized samples were then centrifuged for 25 min at 14,000 rpm, 4 °C, and supernatants were stored at − 80 °C until subsequent analysis. Total protein concentration was determined using a Pierce BCA Protein Assay Kit (Thermo Fisher Scientific, MA, USA). Equal protein concentrations of 20 µg/well were loaded and separated on a NuPAGE™ 10–15% Bis–Tris protein gel (Invitrogen™, MA, USA) and transferred to a PVDF membrane (Bio-rad, USA). iBright™ Prestained Protein Ladder (Invitrogen™, MA, USA) was used to determine the approximate molecular weight of the studied proteins. The membrane was then blocked with 5% non-fat milk prepared in Tris-buffered saline/Tween®20 (TBS-T) for 90 min at RT, followed by incubation with anti-GFAP (1:1000, Merck Millipore, MA, USA), anti-NF-L (1:500, Cell Signaling Technology, MA, USA), or anti-MBP (1:1000, Abcam, UK) primary antibody at 4 °C overnight. Subsequently, the membrane was washed four times with TBS-T and incubated with goat anti-rabbit IgG HRP conjugated (1: 10,000, Santa Cruz Biotechnology, TX, USA) or goat anti-mouse IgG HRP conjugated (1:10,000, Chemicon International, Inc., CA, USA) secondary antibody for another 90 min. Following TBS-T washing, protein bands were visualized using the enhanced chemiluminescence substrate (Thermo Fisher Scientific, MA, USA) and scanned by the Fusion FX Imaging system (Vilber, France). After a short incubation with Restore™ Plus Blot Stripping Buffer (Thermo Fisher Scientific, MA, USA), the membrane was re-used for application of anti-β-actin HRP antibody (1:10,000, Abcam, UK), which served as a sample loading control and normalization protein. The optical density of protein bands was quantified using Quantity One 4.6 Software (Bio-Rad, USA). Partial values were calculated as the ratio of the optical density of the examined protein to β-actin, and final results were expressed as mean ± SEM.

### Immunohistochemical Analysis

The results obtained by Western blot analysis were supplemented by immunohistochemical labeling to localize observed proteins. Fixed spinal cords were cryoprotected and sectioned in Leica CM1850 cryostat to 16 µm thick transverse sections in the same manner as above (Fig. 1). The serial and consecutive sections were then placed on SuperFrost microscope slides and pre-treated in 0.3% PBS with Triton buffer (PBS-T) for 10 min at RT. After subsequent blocking in 5% normal goat serum prepared in PBS for 90 min at RT, sections were incubated with relevant primary antibody overnight at 4 °C. Reactive gliosis was detected with anti-GFAP (1:500, Merck Millipore, MA, USA) antibody, and myelinated neural axons were visualized by anti-NF-L (1:500, Cell Signaling Technology, MA, USA) and anti-MBP (1:500, Abcam, UK) antibodies in colocalization. A day later, sections were washed 4 × 5 min in 0.3% PBS-T and incubated with anti-mouse FITC-IgG (1:200, Jackson ImmunoResearch Laboratories, Inc., USA) and anti-rabbit Rhodamine Red™-IgG (1:400, Jackson ImmunoResearch Laboratories, Inc., USA), respectively, for 90 min at RT. After incubation, spinal sections were washed 4 × 5 min with 0.3% PBS-T and rinsed in distilled water. Fluoromount mounting medium (Serva Serving scientist, Germany) was then used to adhere the cover-slips to the microscope slides. Immunohistochemically-labeled spinal sections were then visualized and digital images captured using the Olympus BX51 fluorescence microscope (Olympus Life Science, Tokyo, Japan) at ×20 magnification.

### Statistical Analysis

All obtained data were statistically analyzed using GraphPad Prism software version 6.01 (La Jolla, CA, USA) and the results expressed as mean ± SEM. Ordinary one-way analysis of variance (ANOVA) with multiple comparison using the Holm–Sidak method (*p* value ˂ 0.05) was used to determine the level of significant statistical differences among control and experimental groups.

## Results

### Spontaneous Locomotor Recovery

**Fig. 3:**
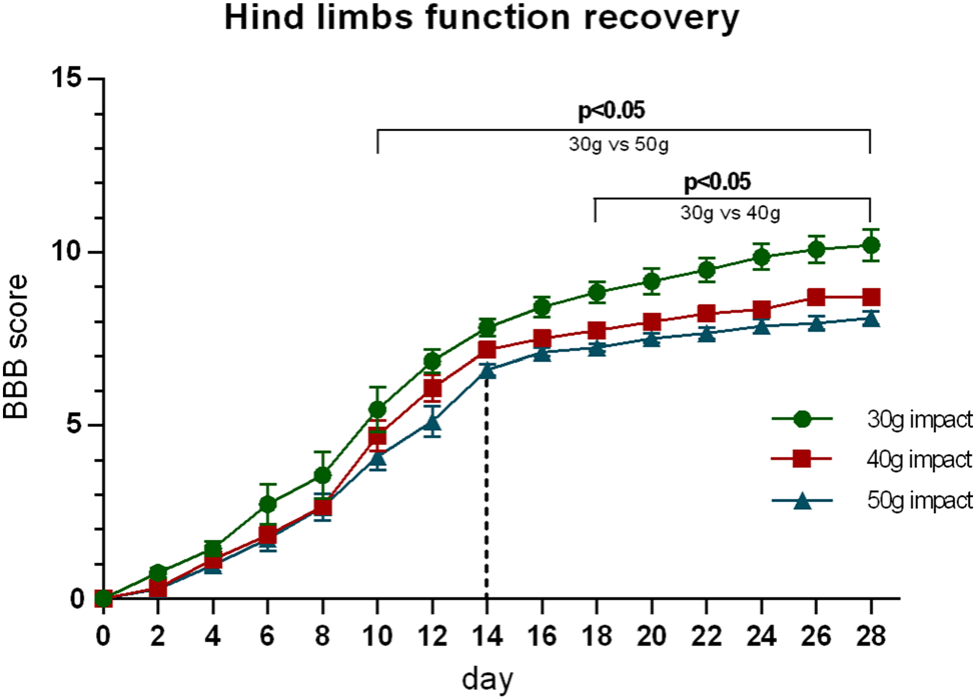
Progress of spontaneous locomotor recovery of hind limb function during the 28 days after SCI with different severity. It can be divided into a *rapid recovery phase* (up to the 14th day) and a *slow recovery phase* (from the 14th day to the end of the survival period). Values are expressed as mean ± SEM of 17 experimental animals per group

Depending on the parameters of initial impact, its duration and spinal segmental level, spinal cord trauma may lead to various temporary, as well as permanent, neurological dysfunctions that deleteriously affect pathways involved in physiological motoric and autonomic functions. Immediately after spinal cord compression, experimental animals displayed a total hind limb paralysis. Spontaneous recovery of locomotor hind limb function, analyzed by the BBB rating scale over 28 days, produced the sigmoidal curves (Fig. 3). Their course can be divided into two phases: a period of accelerated locomotor recovery within the first 14 days (*rapid recovery phase*), followed by slow progress between 14 and 28 days (*slow recovery phase*). Generally, the progress of locomotor recovery after 30 g compression was significantly better from the 10th day (compared to 50 g compression), and from the 18th day (compared to 40 g compression) until the end of the monitored period. On the contrary, the recovery progress after 40 g and 50 g compression was almost identical for the initial 8 days, but slightly and non-statistically different for the rest of the post-traumatic period. At the end of the monitored period, the final mean BBB score after 30 g compression was 10.4 points, which corresponds to plantar stepping with occasional weight bearing. The improvement of locomotor function after 40 g and 50 g compression was more moderate and stabilized at 8.8 and 8.1 points, which correlates to sweeping without weight support.

### Body Weight Changes After the SCI

**Fig. 4:**
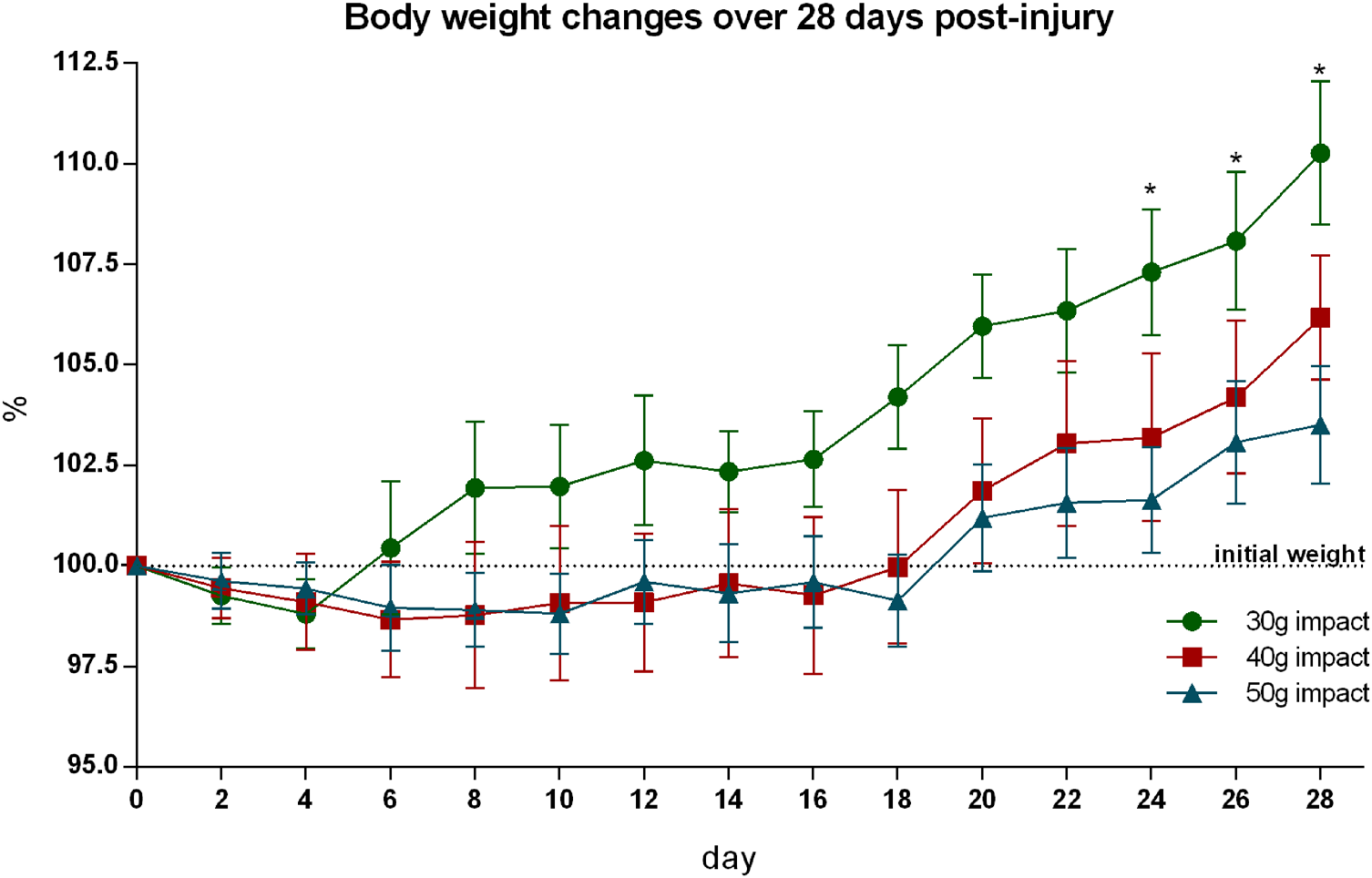
Progress of body weight changes over 28 days after various severe SCI. Results are expressed as percentage of actual body weight compared to animal’s initial weight and are described in the corresponding graph as mean ± SEM of 17 experimental animals per group. *p < 0.05 compared to initial weight

Post-injury weight loss is a common consequence of traumatic SCI. Therefore, body weight during post-traumatic survival was recorded to assess the general health of the experimental animals with the SCI. After a short initial decline, the average body weight after 30 g compression reached pre-injury values on the 6th day (Fig. 4). The mild post-traumatic body weight drop after 40 g and 50 g compression persisted much longer: 18 and 19 days, respectively. A slow and gradual increase of body weight was subsequently noted. The final body weight gain at the end of the survival period negatively correlated with the compression force (i.e., lower body weight gain after more severe compression) and was within the range of 2.5–10% of initial weight.

### Neurogenic Bladder Dysfunction and Post-traumatic Hematuria

**Fig. 5:**
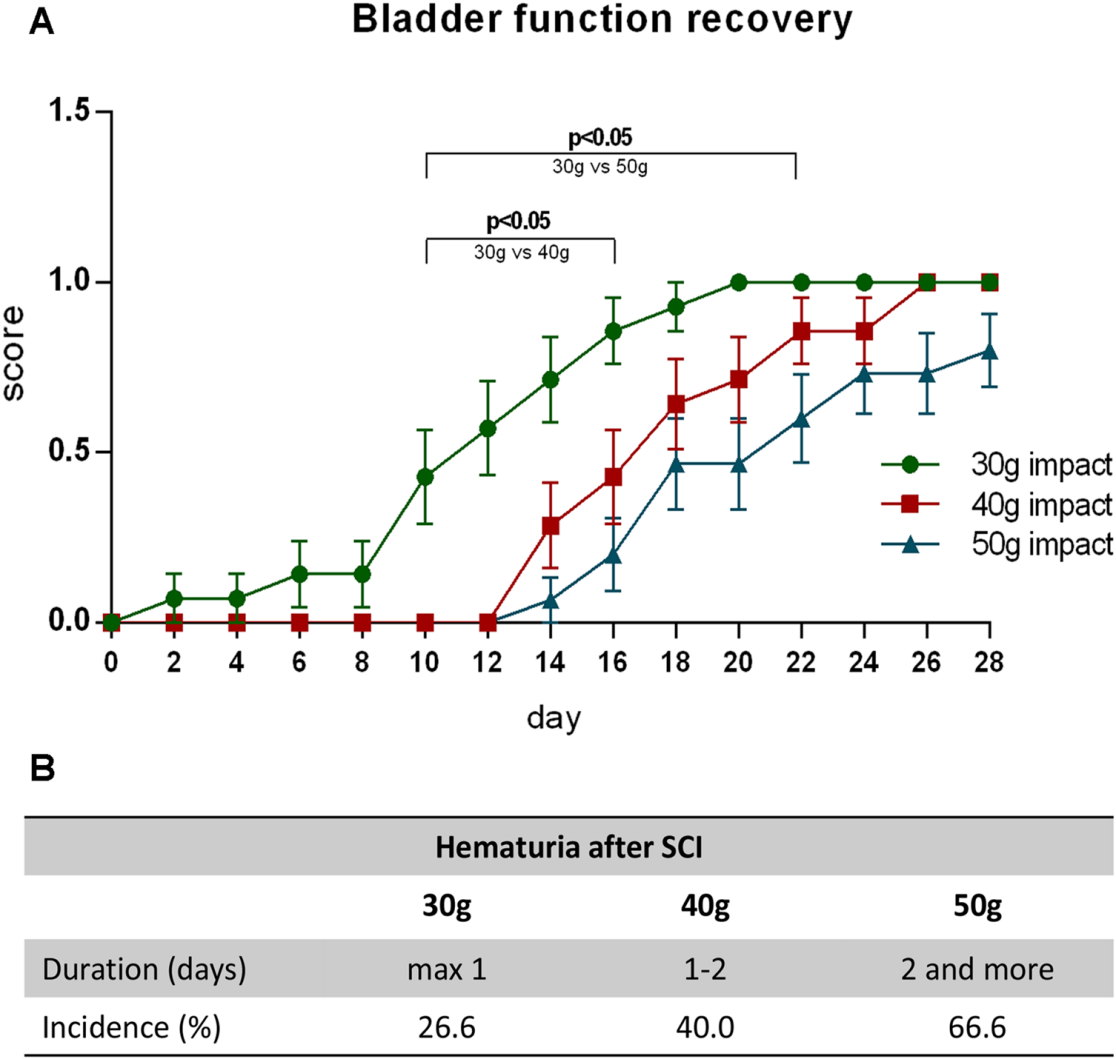
Progressive voiding function recovery after SCI. **a** Graph describing the continuous voiding function regeneration after early bladder areflexia occurred in spinal shock period (0—complete loss of voiding function, 1—physiological bladder function). Values are expressed as mean ± SEM of 17 experimental animals per group. **b** Incidence and duration of hematuria after various SCI

Besides the locomotor disability, complete voiding dysfunction was observed immediately after the compression injury in all experimental SCI groups. Therefore, the bladder was manually emptied daily until the voiding function recovered. Progressive recovery of voiding after 30 g spinal cord compression was detected until the 20th day, when controlled urination was recorded in all experimental animals (Fig. 5a). No signs of voiding recovery were initially seen in the 12 post-traumatic days following 40 g and 50 g compression. Thereafter, a progressive recovery of bladder emptying was detected in both experimental groups. Controlled urination after 40 g compression was stabilized from the 26th post-injury day. However, following the 50 g compression injury, the complete physiological voiding function was only observed in 80% of experimental rats at the end of the monitored period.

Hematuria, which may occur rapidly following traumatic SCI, was observed in all experimental groups. The presence of blood in the urine was observed to be the most extreme on day 1 after 30 g compression, on days 1–2 with an increase of compression weight to 40 g, and persisted for more than 2 days after 50 g compression (Fig. 5b). The incidence of hematuria was 26.6%, 40.0%, and 66.6% respectively; similar to its duration, it was evidently compression force-dependent.

### BSCB Disruption

**Fig. 6:**
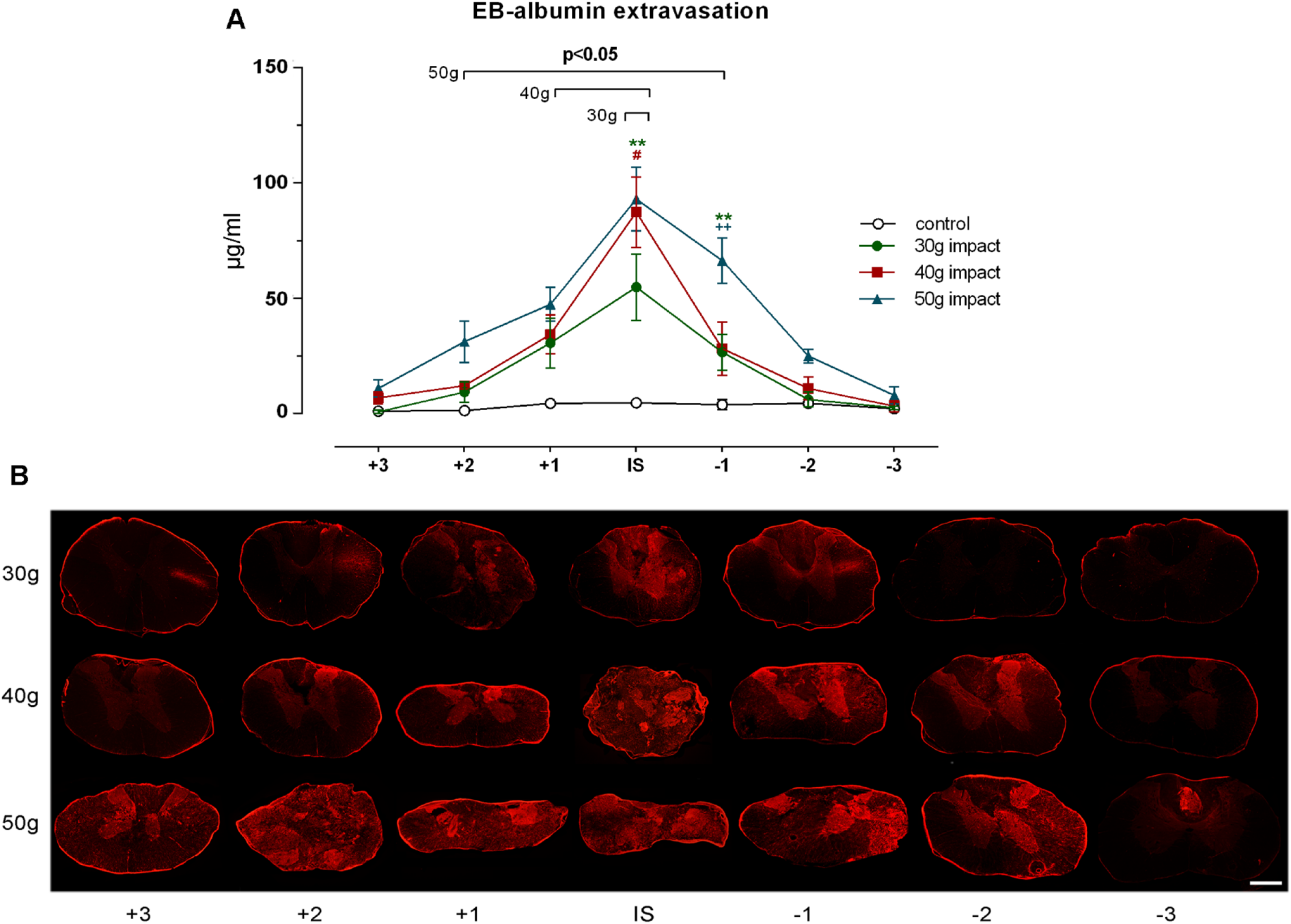
Disruption of BSCB integrity after various compression forces, determined in each segment along the lesioned spinal cord. **a** The concentration of Evans blue dye extravasated into spinal cord parenchyma detected by spectrofluorometric analysis. Values are expressed as mean ± SEM of 4 animals per group. **b** Representative in situ detection of extravasated Evans blue dye in 25 µm thick transverse sections taken from the central region of each analyzed segment. Scale bar 600 µm

The BSCB permeability following traumatic SCI was assessed by intravenous administration of Evans blue dye which rapidly binds to serum albumin, and its extravasation into the spinal cord parenchyma indicates BSCB breakdown. As expected, no spectrofluorometrically detectable concentration of high molecular complex EB-albumin was measured in uninjured spinal cords (Fig. 6a). One day following gradated 30 g, 40 g and 50 g spinal cord compression, an altered BSCB permeability allowed the leakage of stable EB-albumin complex into the spinal cord parenchyma, peaking in the lesion epicenter, where the highest values were measured as follows: 54.8 ± 14.4 µg/mL, 87.4 ± 15.4 µg/mL and 93.0 ± 13.8 µg/mL, respectively. A gradual decline of the leakage was found in the cranio-caudal direction from the lesion epicenter. Interestingly, despite a marked difference in lesion site after compression with 30 g and 40 g weights, an almost similar extent of BSCB disruption was detected in the three cranial as well as the caudal segments. Although the increase in compression weight to 50 g did not cause more pronounced leakage in the epicenter, greater BSCB permeability was seen in the analyzed cranio-caudal segments. A statistically significant extravasation of EB-albumin complex was localized only in the lesion center (3 mm segment) after 30 g compression, but increasing the compression weight to 40 g or 50 g markedly extended the barrier leakage over two (6 mm) or four (12 mm) spinal cord segments.

**Fig. 7:**
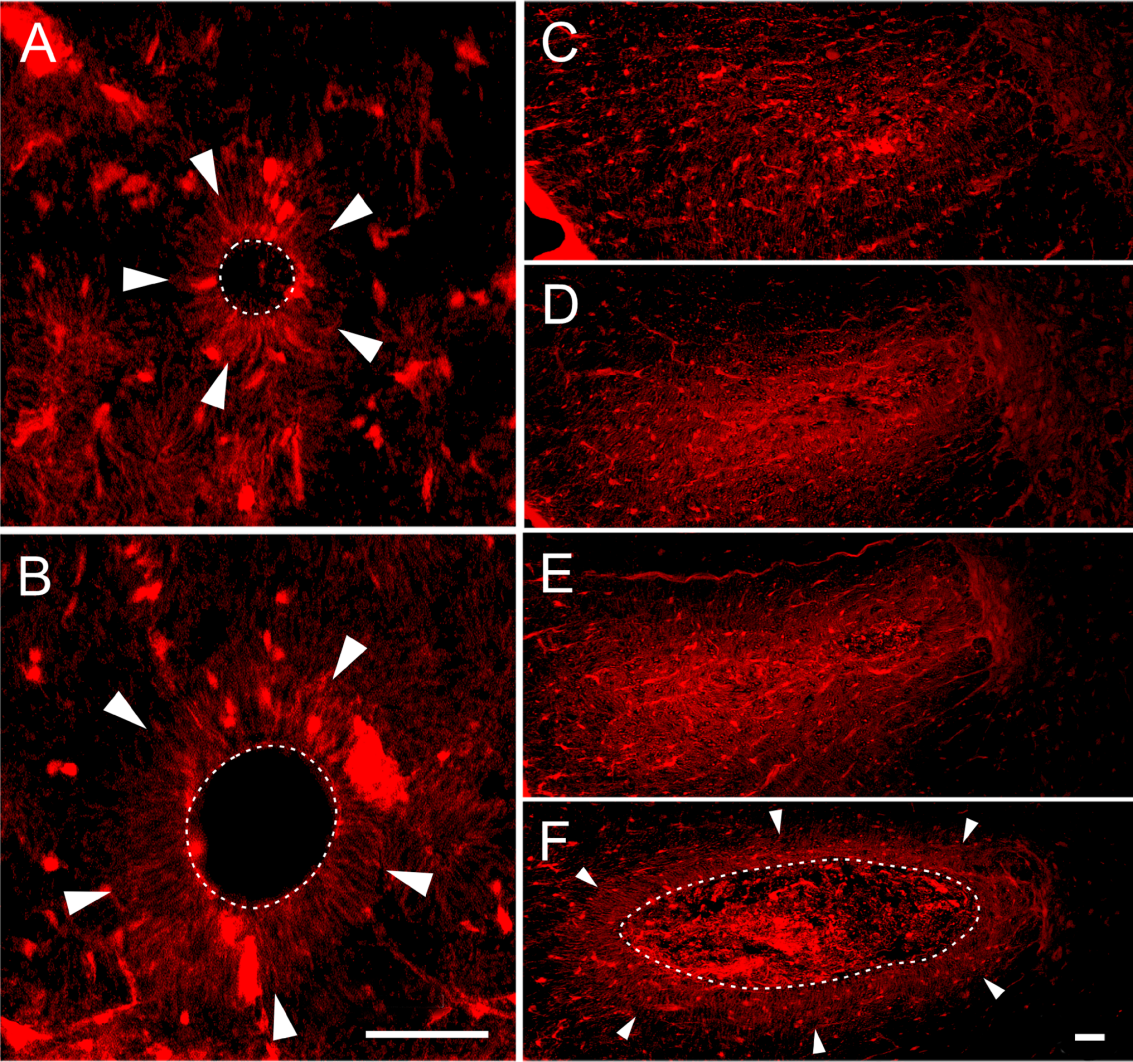
EB-albumin leakage in the intramedullary microcyst (**a**, **b**) and large cystic cavity (**c**–**f**), occurring post-traumatically within the white matter. The fluorescent images represent consecutive sections at 25 µm intervals (microcyst), and at 1 mm intervals (large cyst). Radially and centrifugally oriented leaking of EB-albumin (arrowheads) can be seen within both microcyst and large cyst. Scales represent 20 µm

Since Evans blue can fluoresce with an excitation peak at 470 nm and 540 nm and an emission peak at 690 nm, its extravasation was observed in situ in transverse sections taken along the lesioned spinal cord (Fig. 6b), providing a spatial profile of BSCB disruption after the SCI. Similar to spectrofluorometric analysis, there was no observable leakage of EB-albumin within parenchyma in uninjured spinal cords (data not shown). Overall, the evident fluorescence signal representing extravasated EB-albumin in spinal cord parenchyma after 30 g compression was observed in the lesion epicenter and slightly in the adjacent segments, predominantly in central gray matter and dorsal and lateral white matter columns, which are directly affected by dorsally applied mechanical impact. The barrier leakage in both gray and white matter extended cranio-caudally from the lesioned site with increased compression force, suggesting that the degree of extravasation and its longitudinal extension are directly related to the severity of the initial traumatic insult. We noted that the impaired BSCB was localized in spinal cord tissue adjacent to the cystic cavitation as it is well demonstrated in the dorsal column of the -3 region after 50 g compression. More detailed observation of the trauma-damaged spinal cord revealed a radially and centrifugally oriented EB-albumin leakage from microcysts and larger cavities found within the dorsal and lateral columns of white matter (Fig. 7). The presence of fluorescent cellular structures was evident nearby large cavities, as demonstrated in Fig. 7c.

### The Quantitative Assessment of Histopathological Changes in Gray and White Matter

**Fig. 8:**
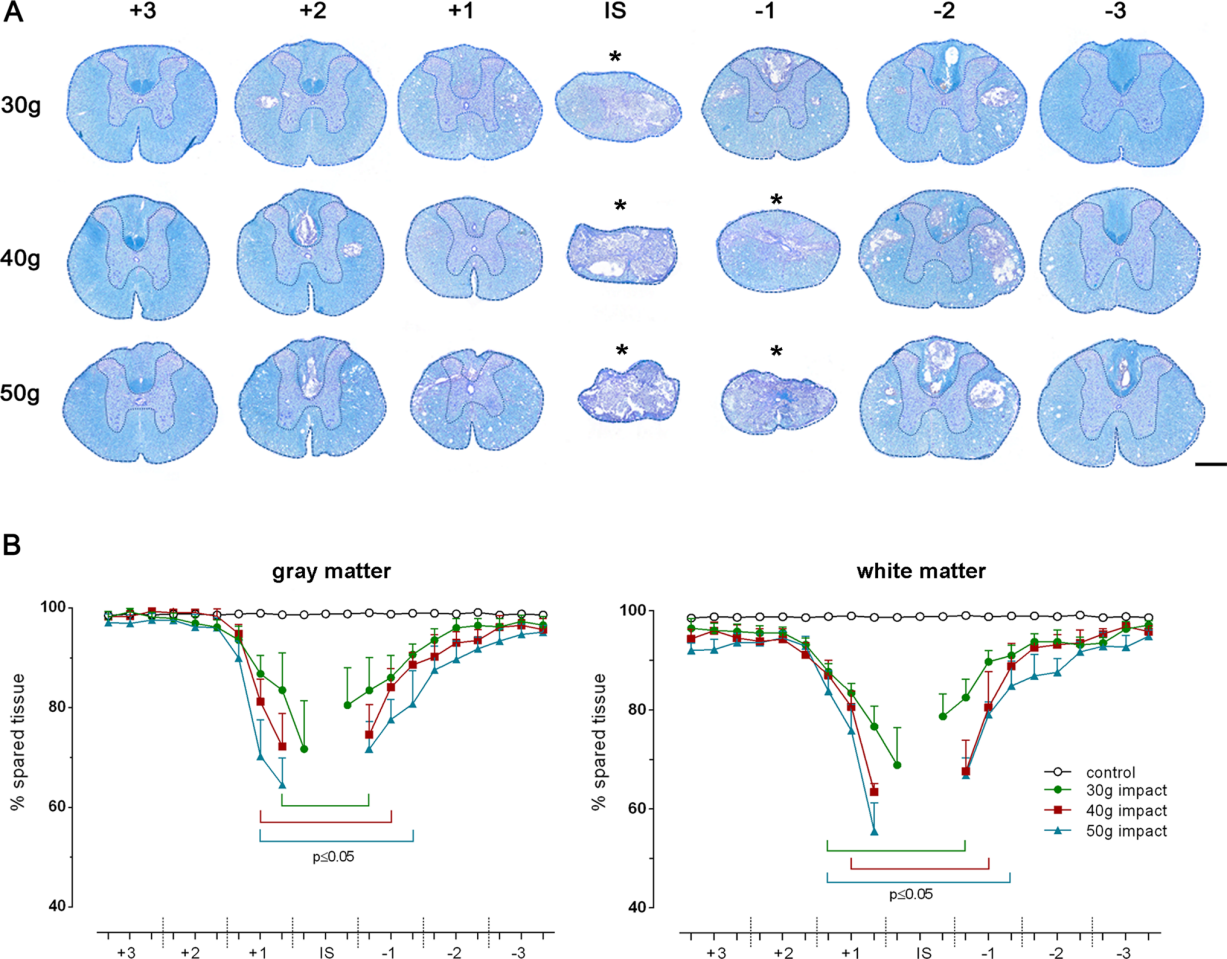
Development of secondary tissue damage in white and gray matter on the 28th day after different compression-induced SCI. **a** Representative transverse sections taken from the central region of each segment stained with LFB/CV demonstrating tissue damage following 30 g, 40 g and 50 g compression. Indistinguishable white and gray matter area in and close to the injury site (IS) is labeled by an asterisk (*). Scale bar 600 µm. **b** Graphs representing the quantification of spared gray (left) or white (right) matter in each region, at 1 mm intervals along the control and variously lesioned spinal cord. Individual values are expressed as mean ± SEM of 10 experimental animals. *p* < 0.05 compared to control

The precise detection of pathological tissue changes after the compression injury was carried out by histological analysis on transverse spinal sections at 1 mm intervals, stained with LFB for myelin, combined with CV for neuronal Nissl substance. The compression force-dependent progress of histopathological changes after the SCI clearly demonstrated the massive degradation of spinal cord tissue, observed as tissue shrinkage predominantly in the injury epicenter (Fig. 8a). Therefore, the initial analysis of histological sections from intact spinal cords was performed in a similar way to the method previously documented (Fedorova and Pavel 2019), in order to a calculate correction factors for both gray and white matter regions along the spinal cord, as summarized in Table 1. The correction factors allowed the estimation of the pre-injury area in the trauma-lesioned spinal cord, which was necessary for the calculation of the spared tissue amount, expressed as a percentage of the total cross-sectional area in a specific region, as briefly described in the Material and Methods section.

Our previously published results showed that the spinal cord tissue loss after gradated (30 g, 40 g and 50 g) compression is mostly evident in the lesion epicenter and progressively declines in the cranio-caudal direction when spinal cord tissue was taken as a whole (Fedorova and Pavel 2019). The degree of tissue degradation was clearly dependent on the initial compression force and negatively correlated with the final BBB locomotor score. Our current experiments were aimed to quantify the gray and white matter lesion along the lesioned spinal cord for the purpose of clinicopathological correlation. A similar course of tissue degradation was noted after 28 post-traumatic days, when two major and functionally different components of the spinal cord were analyzed (Fig. 8b). However, central gray matter surrounded by white matter in the lesioned area were indistinguishable within the range of 2 mm after 30 g injury and 4 mm after 40 g and 50 g compression. Less than 73%, 72%, and 65% compact gray matter and 69%, 63%, and 55% compact white matter was spared in the lesion site, corresponding to 30 g, 40 g and 50 g compression, respectively, representing the minimal values after differentiating two major components of the spinal cord in histologically damaged sections. The statistically significant reduction of gray matter extended over 4 mm (30 g compression), 6 mm (40 g compression) and 7 mm (50 g compression), but affected white matter even more (over 6 mm, 6 mm, and 8 mm, respectively). The presented results suggest that a more prominent degradation of white matter than gray matter occurs in our compression model of the SCI. A rapid degradation of either gray or white matter can be noted in the first cranial segment (+ 1), while gradually decreasing tissue loss was seen within the caudal segments. This suggests that the neurodegenerative effects of secondary injury after spinal cord compression extended more caudally from the primary lesioned area. The obtained histopathological results corresponded with the locomotor outcomes measured at the end of the survival period.

**Fig. 9:**
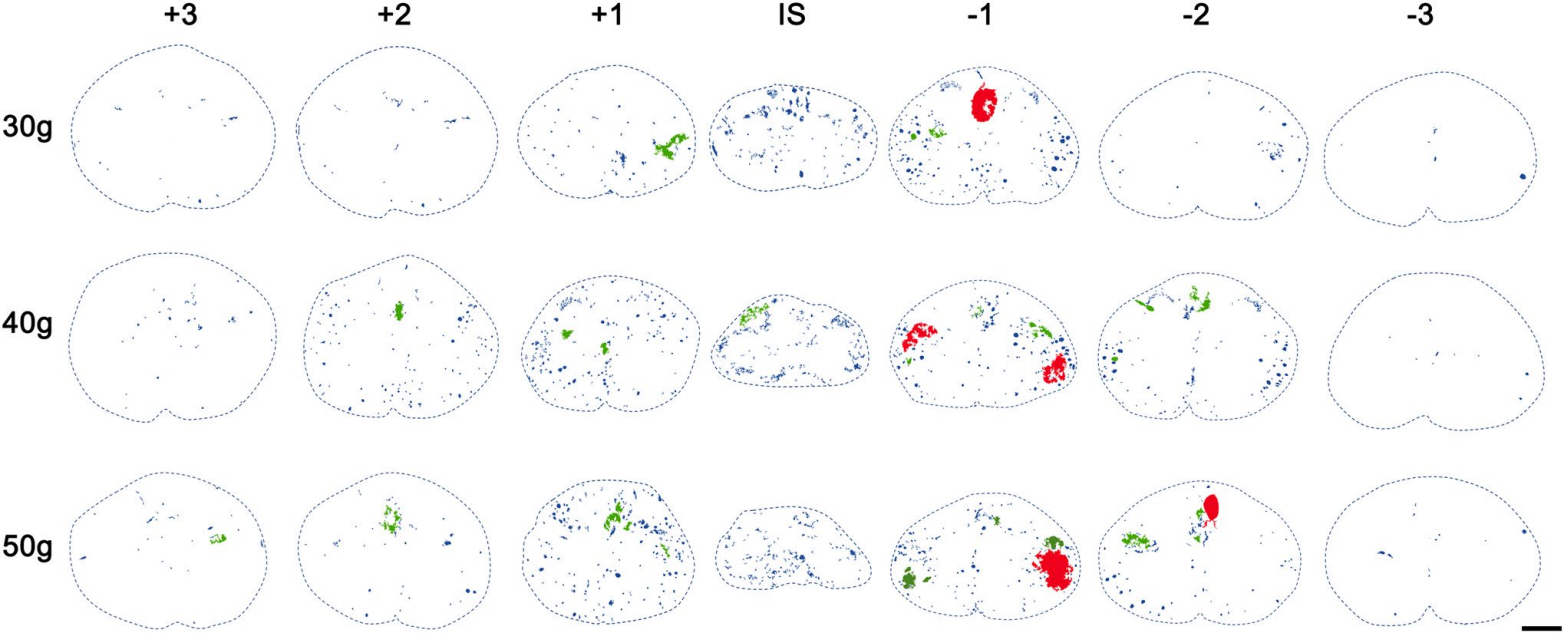
An illustrative demonstration of numerous cystic cavities occurring in transverse spinal cord sections on the 28th post-traumatic day after SCI with different compression weights. Representative pictures demonstrate the presence of small (blue—200–5000 µm^2^), medium (green—5000–50,000 µm^2^), and large (red—over 50 000 µm^2^) cavities in spinal cord sections taken from the central region of each segment. Scale bar 600 µm. *IS* injury site

**Fig. 10:**
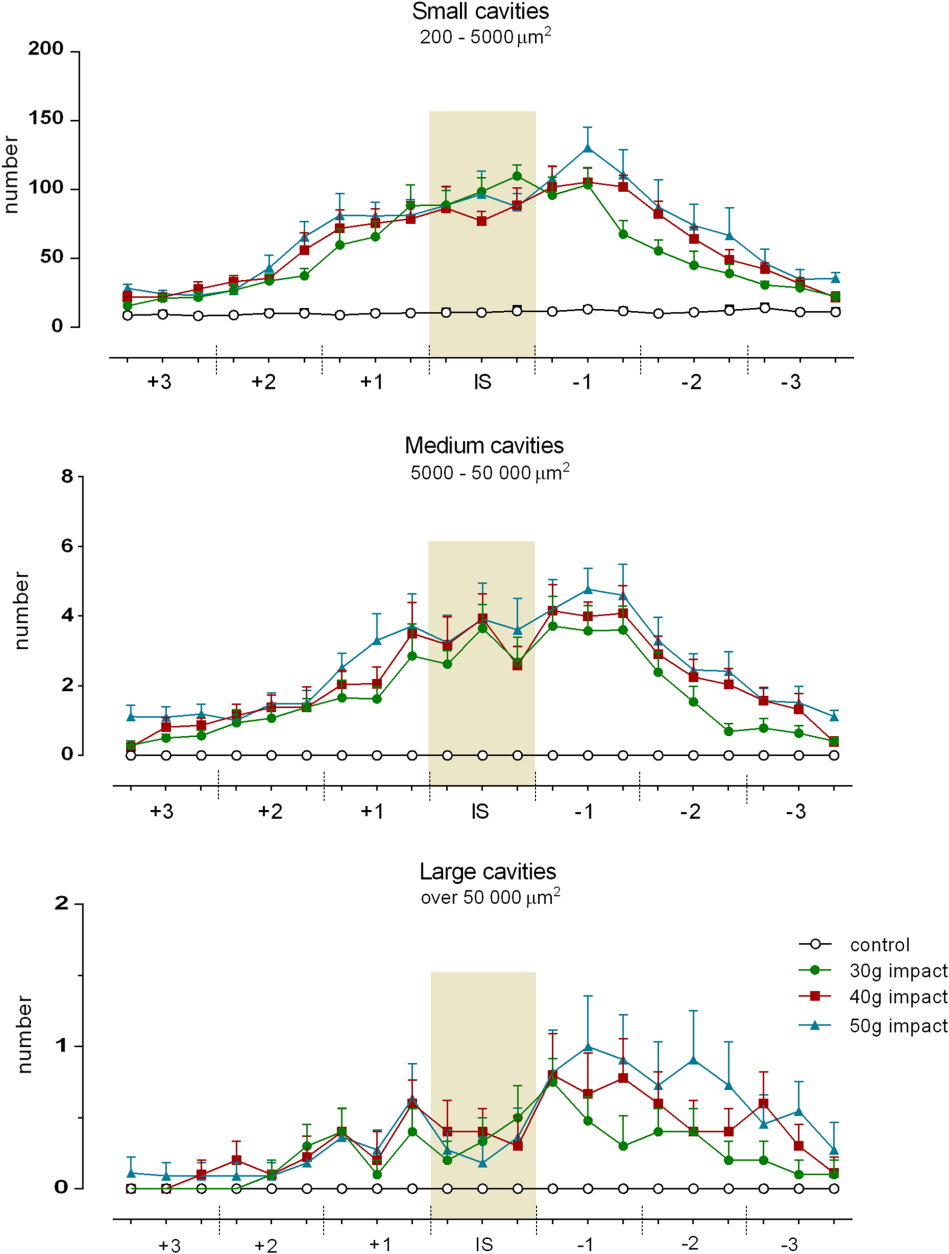
Numbers of cavities classified into three groups according to actual size, automatically measured in each region at 1 mm intervals along differently damaged spinal cords. Values are expressed as mean ± SEM of 10 experimental animals. The injury site (IS) is highlighted

The measured neural tissue loss after experimental SCI was largely due to the formation of microcysts and cystic cavities that occurred within the lesioned spinal cord. Representative images of injured spinal cords after 30 g, 40 g and 50 g compression (illustrated in Fig. 9) clearly demonstrate the presence of multiloculated cystic cavities of various size, formed mainly in the injury epicenter and adjacent segments. Overall, the number of small cavities (200–5000 µm^2^) markedly predominated over the medium (5000–50,000 µm^2^) and large cavities (over 50,000 µm^2^) in all injured and analyzed spinal cords (Fig. 10). Regardless of size, their most frequent occurrence was detected in caudal segments from the lesion site. Small cavities occurred within both gray and white matter regions, but the prevalence of medium and large cavities was observed exclusively in the dorsal and lateral columns of white matter, and predominantly in the caudal direction from the injury site. Large cystic cavities, representing a major constituent of post-traumatic spinal cord tissue reduction, were mainly localized in dorsal columns after 30 g dorsally applied compression; however, they became more prevalent in lateral columns with increasing compression force (Table 2).

**Table 2:** Occurrence of large cystic cavities (over 50,000 μm^2^) forming in white matter columns 28 days after different compression weight applied dorsally on Th9 spinal cord segment

		30 g	40 g	50 g
Cystic cavities (%)	Dorsal column	60	38	35
	Lateral column	40	62	65
	Ventral column	0	0	0

### Post-traumatic GFAP Expression, Myelin and Neurofilament Degradation

**Fig. 11:**
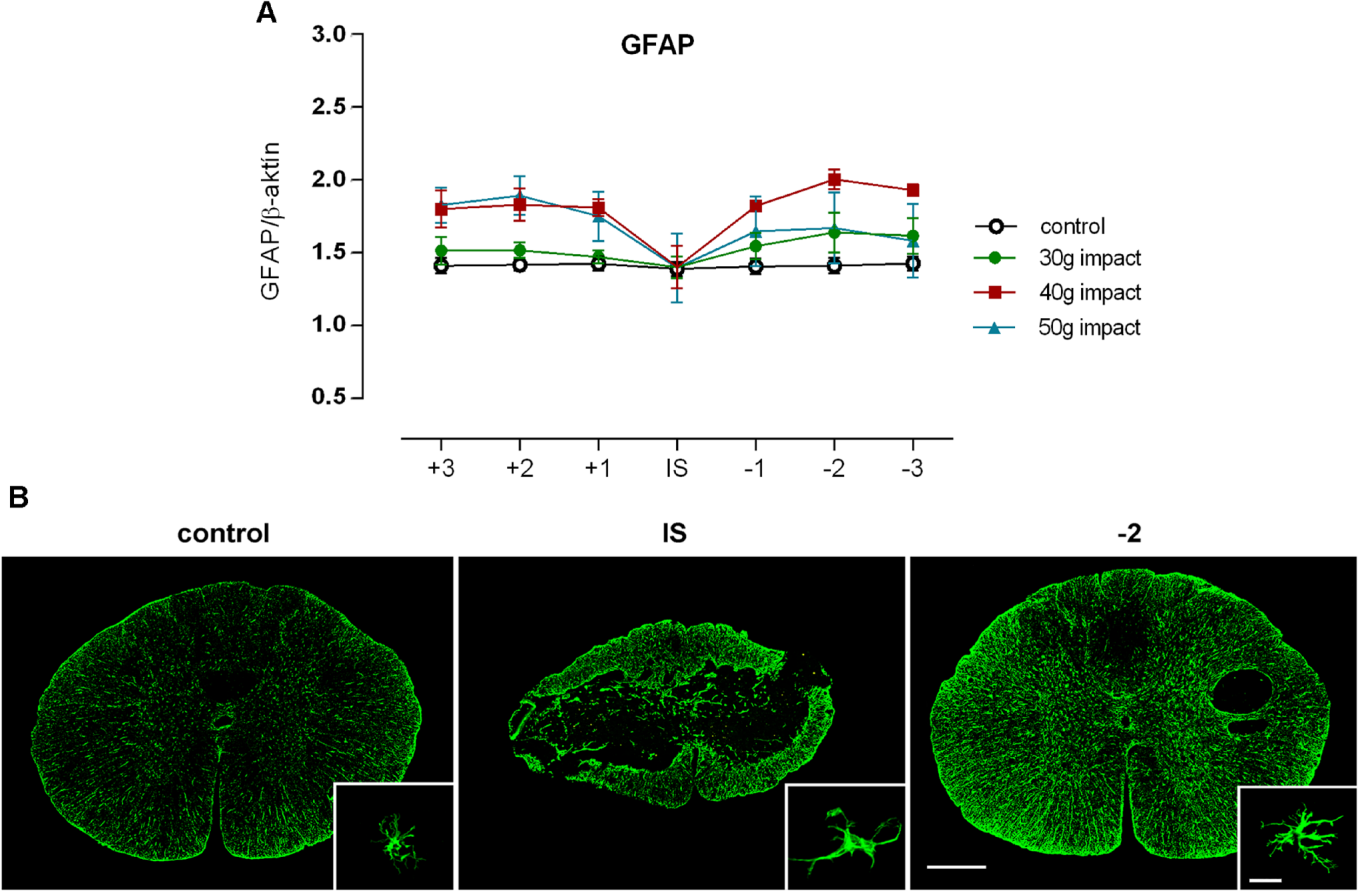
Expression of GFAP, considered as a highly specific marker of astroglial activation, following different spinal cord compression applied dorsally. **a** Graph showing the cranio-caudal changes in GFAP expression from injury site measured in 3 mm spinal cord segments. Values are expressed as mean ± SEM of 5 experimental animals. **b** Immunofluorescence labeling demonstrating GFAP localization from three differently lesioned spinal cord regions. Scale bar 500 µm and 20 µm

Histopathological changes within the injured spinal cord are commonly accompanied by biochemical alterations of glial and neuronal structural proteins such as GFAP, which is an intermediate filament protein primarily expressed in astrocytes, and neurofilaments and MBP, which are the integral components of basal axonal architecture. Since GFAP expression is upregulated by activated astrocytes and structural components, such as neurofilaments and MBP, are degraded following CNS trauma, these were used as sensitive markers for SCI, potentially indicating the severity, as well as the extent, of the damage. The expected upregulation of GFAP expression was detected after different SCIs in all analyzed cranial and caudal segments, except the injury epicenter, where measured out values did not markedly exceed basal levels (Fig. 11a). This is not surprising, because massive tissue degradation involving neuronal and glial cells was demonstrated in this primary lesioned area. A cranio-caudal upregulation of GFAP correlated with the compression force when 30 g and 40 g compression were applied on the Th9 spinal segment. However, further elevation of the compression weight to 50 g did not proportionally increase the GFAP levels in cranial, but mainly in the caudal segments, where protein downregulation was most probably detected due to more prominent tissue loss and the presence of cystic cavities, which were mainly large. Extensive synthesis of GFAP was achieved by activated astrocytes in a process known as reactive astrogliosis. After the SCI, naïve astrocytes became activated and exhibited characteristic morphological features, such as the hypertrophy of cell bodies and cytoplasmatic process extension, as can be seen in Fig. 11b. Such astroglial morphology was detected in the areas surrounding the lesion core. Scar-forming and overlapping astrocytes were found mainly at the lesion border.

**Fig. 12:**
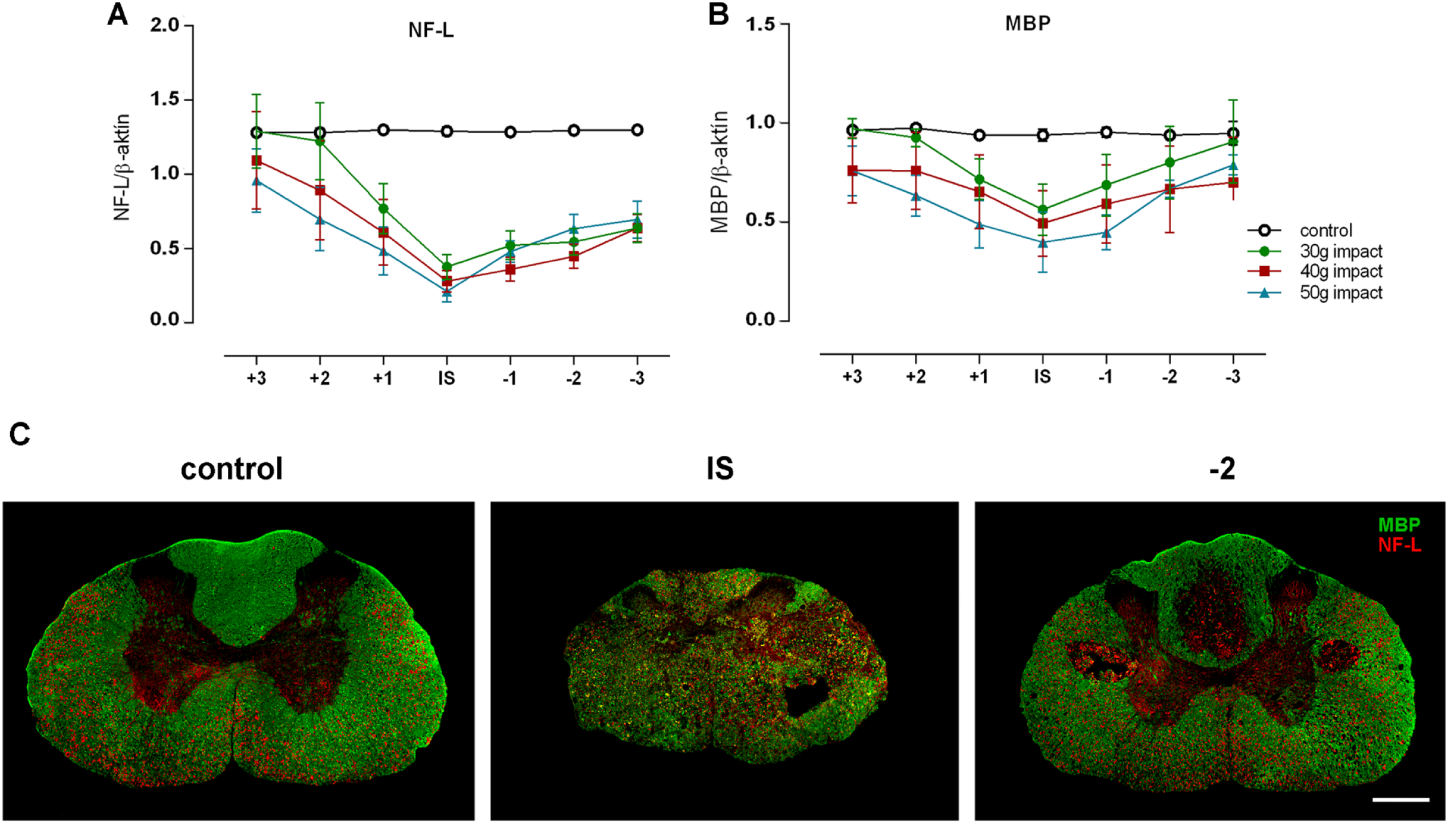
Neuroaxonal injury following compression-induced SCI, demonstrated by degradation of NF-L and MBP. The graphs show the progress of NF-L (**a**) and MBP (**b**) degradation in terms of the cranio-caudal extent from the lesion site. Values are expressed as mean ± SEM of 5 experimental animals. **c** Immunofluorescence labeling demonstrating a regional loss of NF-L and MBP within the lesioned spinal cord. Scale bar 500 µm

Similarly, quantitative analysis of NF-L in trauma-lesioned spinal cord revealed its most profound degradation in the spinal cord segment involving the lesion epicenter (Fig. 12a). After 30 g compression, the degradation gradually weakened in the cranial direction, and the NF-L level in the second (+ 2) segment almost reached the control values. The compression force-dependent progress of NF-L degradation was indicated in this direction after more severe trauma. A slow gradual decrease in the degradation of the neurofilament was determined caudally in all experimental groups, which markedly extended over the analyzed area, suggesting the more profound degradation of structural axonal components below the site of primary injury. Since axonal myelination, and hence a compact myelin sheath, can markedly promote neural transmission, MBP was also analyzed. Longitudinal extension of myelin loss, commonly peaking in the lesion site, was dependent on the severity of the initial trauma and extended cranially and caudally over the analyzed area after 40 g and 50 g compression (Fig. 12b). Double fluorescent immunolabeling showed that the MBP immunoreactivity was abundantly distributed throughout the white matter columns of the intact spinal cord, where myelin rings encircled neurofilament-immunopositive axons (Fig. 12c). Four weeks following the injury, a reduction of either individual myelin rings or axons, as well as myelinated axons, was evidently seen at the site of the primary mechanical insult and continued to a lower extent, mainly caudally, outside the analyzed segments, as revealed by quantitative WB analysis.

## Discussion

Extensive analysis of human cases of spinal cord trauma have revealed that the primary mechanism of injury is usually the direct-mechanical compression of spinal cord parenchyma resulting from the dislocation of a fractured vertebra or disc (Kakulas 1999). A physical deformation of the spinal cord tissue transmits compressive forces on neuronal and glial cell bodies and their projections, as well as on blood vessels, and initiates a cascade of pathological events involving a systemic and local dysfunction of the vascular network (including neurogenic shock, hemorrhage, and an impairment of blood flow autoregulation), edema formation, elevation of intraspinal pressure, BSCB breakdown, extensive inflammatory response associated with the elimination of necrotic debris, and the development of cysts/cavities and syrinx, etc.

The final damage caused by spinal cord compression depends on both the weight and duration of the compression. Thus, our primary goals were to specify these two key parameters to optimize the severity of the injury and to ensure a low degree of spontaneous recovery. Since it was demonstrated that a 20 g compression lasting 20 or even 40 min did not induce a severe and long-term impairment of hind limb function (Hamamoto et al. 2007), we started our experimental compression at a 30 g weight. Preliminary results showed that the 30 g compression maintained for 5–10 min produces only a transient paraplegia with a very high level of spontaneous locomotor recovery (not shown). Further experiments confirmed the results of a previously published study (van Gorp et al. 2013) demonstrating that the spinal cord compression has to be maintained for a minimum of 15 min to produce a reproducible deficit in motor function of the lower extremities and still be sufficient to reveal potential therapeutic effects. Increasing of the compression weight to 40 g only slightly worsened the motor function, and its further increase by 10 g did not even have a significant effect. Generally, the course of hind limb function recovery over 4 weeks in our SCI weight-compression model can be divided into two distinctive phases: (1) *a rapid recovery phase* characterized by accelerated recovery of motor function within 14 days, (2) *a slow recovery phase* characterized by a limited recovery and relatively stable functional deficits beginning at 2 weeks after the compression injury.

Taking into account the rate of body weight loss or gain in the evaluation of locomotor function, the body weight of experimental animals was recorded over the recovery period. Non-significant changes of body weight during the rapid recovery phase of locomotor function (lasting up to 2 weeks) compared to initial values, and mild weight gain during the slow recovery period (lasting up to 4 weeks) exclude the possibility that the body weight changes could be responsible for limited functional recovery, especially in the second half of the post-traumatic period. Even if post-injury weight loss is a common consequence of the SCI (Primeaux et al. 2007), the application of spinal cord compression inducing a severe locomotion deficit did not provoke an inability to maintain body weight within normal ranges, which gives evidence of the good general health of trauma-injured animals.

The SCI at the cervical or thoracic level can disrupt a voluntary and supraspinal control of voiding, as well as normal reflex pathways that coordinate the bladder and sphincter function (de Groat and Yoshimura 2006). A spinal cord trauma initially leads to bladder areflexia and complete urinary retention and is then followed by a slow development of automatic urination and neurogenic detrusor overactivity, mediated by spinal reflex pathways (Fowler et al. 2008). However, voiding is still insufficient due to simultaneous contractions of the bladder and urethral sphincter (detrusor-sphincter dyssynergia). Assisted bladder emptying was necessary from the first day after inducing the compression injury. Gradual recovery of voiding was detected from the beginning of the survival period following 30 g compression, but no signs of recovery were seen until 12 days after more severe injuries. Controlled urination of all animals in individual experimental groups was noted approximately 3 weeks after 30 g compression, and on the 26th day after 40 g compression, indicating that recovery of bladder function after the SCI is dependent upon the reorganization of reflex pathways involved in the control of the lower urinary tract (de Groat and Yoshimura 2006). In addition to altered bladder function, early SCI-dependent disruption of bladder uroepithelial integrity causes an increased permeability to urine and urine-borne substances, resulting in cystitis and subsequent hematuria (Herrera et al. 2010). Under normal post-operative care, the presence of blood in the urine was only transient and persisted for a maximum of 4 days. Our present results indicate that the different severity of compression-induced SCI correlates not only with the impairment of hind limb function, but also with the progress and full recovery of the voiding function, as well as the incidence and duration of hematuria.

Post-traumatic neural tissue necrosis and collapsed cavities can lead to a progressive shrinkage of the rat spinal cord after contusion- (Noble and Wrathall 1989) and compression-induced SCI (Fedorova and Pavel 2019), which occurs over time and is evident predominantly in later periods of the injury. Therefore, the assessment of preserved or lost tissue in lesioned spinal cord by the direct measurement of the total cross-sectional area only, without knowing the pre-injury area, may result in an underestimation of tissue loss or an overestimation of tissue sparing. Our recently published quantitative method using correction factors overcomes the issue of post-traumatic spinal cord shrinkage and is relative fast, accurate, and optimally reproducible (Fedorova and Pavel 2019). We demonstrated that no quantitative differences can be detected along the spinal cord in the rat model without this approach, particularly for severe graded injuries. The correction factors allowed the estimation of the pre-injury cross-sectional area occupied by white or gray matter in each spinal region along the lesioned spinal cord, which subsequently resulted in a reliable post-traumatic quantification.

Standard histological analysis of the lesioned spinal cord with LFB/CV allowed differentiation between gray and white matter, and an estimation of lesion size and formation of cysts and cavities. A quantitative histological evaluation of three different injury severities analyzed in serial sections spaced 1 mm apart revealed a marked degradation of both gray and white matter within the lesion epicenter, which extended more caudally from the primary lesioned area. Although differentiation of both spinal cord substances within the lesion epicenter was not properly possible, the degree of both gray and white matter degradation is apparently compression force-dependent and negatively correlates with final functional outcomes. Dorsally applied severe compressions resulted in the most significant damage to central gray matter and to dorsal and lateral white matter columns. Post-traumatic loss of spinal cord tissue was associated with the formation of numerous cysts and cavities. Small-sized (200–5000 µm^2^) cystic cavities occurred in both gray and white matter, but medium (5000–50,000 µm^2^) and large (over 50,000 µm^2^) cavities were observed exclusively in dorsal and lateral columns, predominantly below the injury site. It should be taken into account that the cavity can sometimes collapse (Metz et al. 2000); therefore, their quantification does not have to reflect a real state and can substantially contribute to the spinal cord shrinkage, especially in the lesioned area. Interestingly, large-sized cavities, which represent a major constituent of tissue loss after compression with the lowest 30 g weight, were localized mainly in dorsal columns, but became more prevalent in lateral columns with increasing compression weight. In any case, maximal tissue preservation and prevention of the formation of cystic cavities in these white matter regions may increase the functional outcomes, since the rubrospinal tract, running in the dorsolateral column, and the corticospinal tract in the dorsal column, as well as reticulospinal fibers in the ventrolateral or lateral columns, are involved in the control of voluntary locomotion (Schucht et al. 2002). Much lower drainage of interstitial fluid via perivascular spaces, resulting from a lower vascular density, may partially explain a preferential occurrence of cystic cavities within white matter columns compared to gray matter (Liu et al. 2018).

Although, the spinal cord is naturally well protected and quite tolerant to slow compression and stretching (Young 2002), a rapid or prolonged mechanical deformation can cause devastating tissue damage as a result of mechanical factors from the initial insult. The compression force on the spinal cord produces longitudinal and parabolic movement of spinal parenchyma, which is most intense in the center, as demonstrated on a physical modeling of the spinal cord contusion (Blight 1988). Anisotropic properties and the different elasticity of the white and gray matter (Popovich et al. 2012) are also important dimensional and physiological variables resulting from the spinal cord composition. All of these aspects may substantially affect the impairment of the local vascular integrity of the spinal cord. Vascular damage in human SCIs is extremely rare in extrinsic superficial arteries (Jellinger 1976), but it occurs primarily in the intrinsic (intramedullary) vascular system (Tator and Koyanagi 1997). The most frequently affected by acute compression have been shown to be the central (sulcal) arteries (Koyanagi et al. 1993). Since they represent the most important intramedullary blood source ramified from the anterior spinal artery and centrifugally supplying the substantial majority of gray matter and inner half of the white matter (Martirosyan et al. 2011), their disruption subsequently interrupts the blood supply to the capillary beds and causes extensive ischemia of the spinal cord parenchyma. Our histological analysis in the lesion site supported these findings since the extensive damage of spinal cord parenchyma corresponds very well with the territory supplied by central (sulcal) arteries, whereas the spared neural tissue was commonly seen in an outer rim of white matter supplied by other intrinsic vascular sources.

Generally, the SCI can also lead to a breakdown of the BSCB followed by bleeding and edema, all of which impair normal hemodynamics and cause a spinal cord ischemia as a result of the direct disruption of blood vessels and increased intraspinal pressure. The BSCB breakdown occurs within 5 min of traumatic SCI (Maikos and Shreiber 2007), with maximal leakage during the early acute phase (Whetstone et al. 2003), and it remains compromised even 8 weeks after primary injury (Cohen et al. 2009). Extravasated EB-albumin was primarily seen in the highly vascularized gray matter and also extended cranio-caudally from the lesioned site along white matter columns with increasing compression weight. Detailed microscopic examination of severely damaged spinal cord revealed a radially and centrifugally oriented EB-albumin leakage from microcysts and larger cavities found within dorsal and lateral columns of white matter, as demonstrated in Fig. 7. The presence of fluorescent EB-albumin positive cellular structures was evident in nearby large cavities, suggesting that EB-albumin leaking from these microcysts or cavities, most probably filled with extracellular fluid derived from circulation and/or directly by cerebrospinal fluid, is forced by a pressure-gradient rather than simply by diffusion. These results confirmed a previous experimental study demonstrating the impaired BSCB in the spinal cord parenchyma adjacent to cysts (Josephson et al. 2001). Fluid filled cysts or microcysts occurring in our weight-compression model of severe SCI may represent (1) the development of post-traumatic syringomyelia, characterized by the presence of fluid-filled cysts, also called syrinxes, that can expand over time, resulting in additional compression (Shields et al. 2012), and/or (2) late stage myelomalacia, a “softening” of the injured spinal cord parenchyma whose early stage is most frequently characterized by the presence of edema (Ramanauskas et al. 1989).

The pathophysiology in many compression models is primarily related to the ischemia-induced changes. However, histopathological changes in our weight-compression model inducing a severe injury are characterized by the development of cavities, mainly caudally, below the compression site. This is in contrast to the “pure” ischemia/reperfusion-induced SCI seen in aortic balloon occlusion models, in which a selective loss of inhibitory interneurons was documented in previously ischemia-exposed spinal segments in the absence of cavity formation (Taira and Marsala 1996; Marsala and Yaksh 1994). It was demonstrated that 20 g compression weight is sufficient to reach a complete ischemia of the rat spinal cord at the lower thoracic level (Hamamoto et al. 2007), suggesting that further tissue damage due to an increase of compression weight is not caused primarily by ischemic (vascular) factors, but rather by a mechanical component. Therefore, the weight-compression model used can instead be considered as the contusion/compression model. Anyway, the fact that prolonged compression after the initial impact was necessary clearly demonstrates a critical role of spinal cord ischemia in subsequent neurodegeneration. The majority of gray matter loss following the SCI occurs within hours and is mostly complete by 24 h, but a loss of white matter appears to extend over several post-injury days (Ek et al. 2012). The neurological deficit resulting from traumatic SCI is predominantly caused by the loss of white matter, particularly the long ascending and descending tracts (Medana and Esiri 2003). In addition to direct axonal lesions, neighboring axons not primarily affected by mechanical stress also show signs of delayed degeneration (Lingor et al. 2012).

It was indicated that the white matter is more vulnerable to ischemia than the gray matter in the rat model of SCI (Follis et al. 1993). This statement is supported by the experimental study showing that the ischemic zones resulting from severe spinal cord trauma were especially serious in the white matter area adjacent to hemorrhages in the gray matter (Tator and Fehlings 1991). After acute compression injury in the rat, ischemia began almost immediately, worsened during the first 3 h, and persisted at least 24 h. Besides, a higher vulnerability to the development of vasogenic edema inducing post-traumatic ischemia was observed also in the white matter (Kimelberg 1995). Macroscopic morphological analysis showed that the axons after the SCI remain completely stable within 30 min (Lingor et al. 2012). However on the molecular level, a rapid calcium influx into the axon and its transient axoplasmic elevation within 40 s after traumatic lesion initiate a signaling cascade resulting in the axonal fragmentation. The axoplasmic calcium elevation and subsequent calcium-sensitive processes play a crucial role in axonal pathology since application of calcium channel inhibitors almost completely inhibited acute axonal degeneration (Knöferle et al. 2010). The parts of the axon that are not affected by acute axonal degeneration are morphologically stable for the first 24 to 72 h, but then the distal part undergoes a progressive defragmentation and subsequent removal in process known as Wallerian degeneration (Kerschensteiner et al. 2005). Using in vitro model of chronic white matter degeneration by prolonged hypoxia was detected a prominent and progressive axonal degeneration; however, myelin sheaths surrounding degenerating axons and oligodendrocytes exhibited remarkable resilience to hypoxia (Cui et al. 2020).

The spinal cord areas that are at risk of progressing to infarction following the SCI, but still salvageable if perfused, can expand as compression continues. Experimental study using the rat SCI-stenosis model, which mimics the clinical SCI and concomitant spinal cord compression, indicated that early decompression preserves intact spinal cord tissue, decreases the lesion volume, and improves functional recovery (Shields et al. 2005). Several previous and recent systematic reviews of the human studies concluded that early decompression (within the first 24 h after acute traumatic SCI or even earlier) results in improved neurological outcomes (La Rosa et al. 2004; Fehlings et al. 2012; van Middendorp et al. 2013; Grassner et al. 2016). However, an improved neurological recovery was evident only among cervical SCI patients (Wilson et al. 2017), and it was associated with the early surgical decompression of complete traumatic SCI (ter Wengel et al. 2019). No significant differences in neurological outcomes were proved between early and late surgery in incomplete traumatic SCI at cervical level.

In addition to histopathological analysis, other sensitive markers indicating the severity and extent of the injury were analyzed, such as the GFAP, neurofilaments, and MBP. Similar to a previously published study (Kwiecien et al. 2020), reactive astrogliosis manifested by the GFAP overexpression was still present on the 28th post-injury day, and it can even persist for almost three months. In humans, the extent of reactive astrogliosis is relative mild, and glial scar formation as a dense network of overlapping reactive astrocytes creating an impenetrable barrier is practically never seen (Norenberg et al. 2004). It seems that reactive astrocytes do not create a physical obstacle to the outgrowth of nerve fibers within the lesion site in our experimental SCI model. Only a thin astrogliotic wall was observed surrounding the cysts and cavities, probably representing the final “healing” phase of the necrotic process (Pekny and Nilsson 2005). Nevertheless, axonal regeneration may be physically inhibited by the progressive formation of a fibrotic scar, which consists of extracellular matrix proteins. It was shown that a selective ablation of reactive astrocytes in an experimental stab model in mice leads to a prolonged increase in leukocyte infiltration, a failure of BSCB repair, neuronal degeneration, and increased outgrowth of nerve fibers in injured parenchyma (Bush et al. 1999).

Functional motor deficit resulting from a traumatic SCI is mainly associated with conductive failure of white matter tracts (Fehlings and Tator 1995). After the SCI, there might be remnants of descending pathways that cannot be effectively used for locomotion due to myelin loss or a limited number of axons. Measurement of the NF-L level in segments along the lesioned spinal cord revealed a compression force-dependent fragmentation of the myelin structure proximally and much extensive fragmentation, independent of compression force, distally from the lesion epicenter in the area that exceeded the analyzed segments. This may suggest Wallerian degeneration in both the cranial and caudal direction, but more prominently in the latter direction, away from the impact site, which proceeded into visually compact spinal cord tissue. As the axon progressively dies, the myelin sheath disintegrates. However, primary demyelination is an important factor in experimental SCIs, but is uncommon in human SCIs, where it is sparse and affects only isolated axons (Kakulas 1999). In our SCI model, the extent of myelin sheath fragmentation does not simply follow the anterograde disintegration of irreversibly damaged axons after compression-induced SCI, as it can be seen in distal segments. On the other hand, the axon is capable of surviving without a myelin sheath and may be remyelinated later. However, without additional experiments, the degree of possible remyelination that occurs during this post-injury stage in the analyzed spinal cord segments can only be speculated.

In conclusion, our histological, immunohistochemical, and functional outcomes demonstrate that this experimental model of graded severe SCI through control over the compression force can effectively modify the severity of injury. Given the small changes in physiological (hind limb and bladder function recovery, body weight gain) and histological parameters (degradation of both gray and white matter associated with the formation of cystic cavities, and structural proteins) between 40 g and 50 g compression, and taking account of the animal´s welfare, the 40 g weight can be considered as an upper limit for severe traumatic injury in this compression model. Pathophysiological mechanisms leading to degeneration of both gray and white matter in this model are primarily related to ischemia–reperfusion changes, but increasing the compression weight seems to increase the proportion of the mechanical component. This model of severe compression sufficiently meets the criteria required for an optimal experimental SCI model.
