# Correction to: The Histopathology of Severe Graded Compression in Lower Thoracic Spinal Cord Segment of Rat, Evaluated at Late Post-injury Phase

**DOI:** 10.1007/s10571-021-01149-5

**Published:** 2021-09-17

**Authors:** Jana Fedorova, Erika Kellerova, Katarina Bimbova, Jaroslav Pavel

**Affiliations:** Department of Neurodegeneration, Plasticity and Repair, Institute of Neurobiology, Biomedical Research Center of the Slovak Academy of Sciences, Soltesovej 4‑6, 040 01 Kosice, Slovakia

## Correction to: Cellular and Molecular Neurobiology 10.1007/s10571-021-01139-7

The original version of this article unfortunately contained error in author group.

The given name and family name of all the authors was swapped and published incorrectly. Now, the correct author names are presented in this correction article.

The original article has been corrected.

